# Exceptionally high strain-hardening and ductility due to transformation induced plasticity effect in Ti-rich high-entropy alloys

**DOI:** 10.1038/s41598-020-70298-2

**Published:** 2020-08-06

**Authors:** Rajeshwar R. Eleti, Margarita Klimova, Mikhail Tikhonovsky, Nikita Stepanov, Sergey Zherebtsov

**Affiliations:** 1 Laboratory of Bulk Nanostructured Materials, Belgorod National Research University, Pobeda 85, Belgorod, Russia 308015; 2 National Science Center “Kharkov Institute of Physics and Technology” NAS of Ukraine, Kharkov, 61108 Ukraine

**Keywords:** Engineering, Materials science

## Abstract

Ti-rich body-centered cubic (BCC, *β*) high-entropy alloys having compositions Ti_35_Zr_27.5_Hf_27.5_Nb_5_Ta_5_, Ti_38_Zr_25_Hf_25_Ta_10_Sn_2_, and Ti_38_Zr_25_Hf_25_Ta_7_Sn_5_ (in at%) were designed using bond order (Bo)-mean *d*-orbital energy level (Md) approach. Deformation mechanisms of these alloys were studied using tensile deformation. The alloys showed exceptionally high strain-hardening and ductility. For instance, the alloys showed at least twofold increment of tensile strength compared to the yield strength, due to strain-hardening. Post-deformation microstructural observations confirmed the transformation of *β* to hexagonal close packed (HCP, *α*′) martensite. Based on microstructural investigation, stress–strain behaviors were explained using transformation induced plasticity effect. Crystallographic analysis indicated transformation of *β* to *α*′ showed strong variant selection (1 1 0)_*β*_//(0 0 0 1)_*α*′_, and [1 − 1 1]_*β*_//[1 1 − 2 0]_*α*′_.

## Introduction

As is well known, pure Ti at ambient temperatures has a crystalline arrangement of the hexagonal close packed (HCP, *α*) structure. *α*-Ti, however, transforms into the body-centered cubic (BCC, *β*) allotrope at elevated temperatures, > 882 °C, aided by diffusional processes. Addition of some transition metal solutes (*β*-stabilizers) facilitates the high-temperature *β* phase stable at room temperature. The so-called *β* alloys at ambient temperatures are however, unstable/metastable. Metastable *β*-Ti alloys are referred after their ability to transform through diffusionless shear processes under the externally applied stress. While some metastable *β*-alloys undergo twinning induced plasticity (TWIP) effect^1^, the others exhibit transformation induced plasticity (TRIP) effect^2,3^. *β*-Ti alloys show different forms of phase transformation during deformation. Among them, most notable are *β* to *α*, *β* to *α*′ (HCP martensite) or *α*″ (orthorhombic martensite) and *β* to *ω* (hexagonal)^2^. TWIP or TRIP assisted deformation of *β*-Ti alloys was reported providing high strength and ductility simultaneously, overcoming the strength-ductility trade-off^2–4^. Furthermore, Ti alloys are also well known for their low elastic moduli^5^, low density and high specific strength^6,7^. With regards to such unique properties, *β*-Ti alloys have been considered as potential candidates for biomedical and aerospace applications.

**Figure 1:**
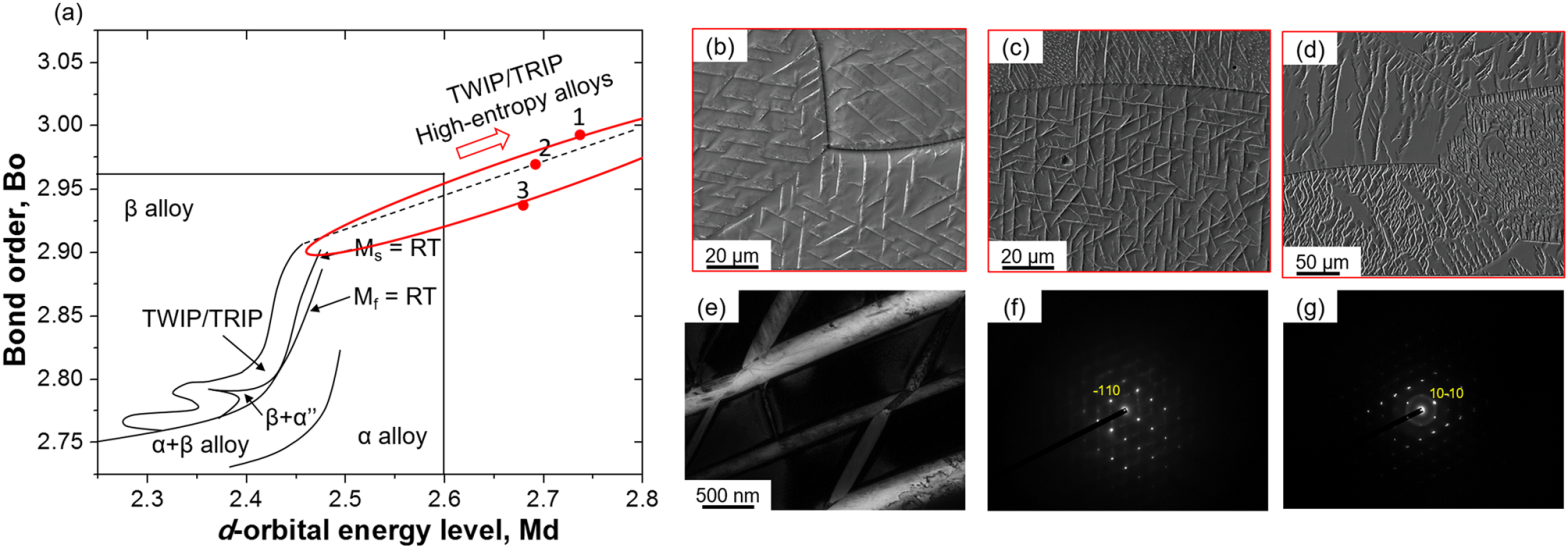
Bo–Md diagram and microstructures of various alloys. (**a**) Schematic illustration of the standard Bo–Md diagram. Broken line indicates the guided reference for alloy design. Red color dots show the selected locations of alloys composed of chemical compositions, 1. Ti_35_Zr_27.5_Hf_27.5_Nb_5_Ta_5_, 2. Ti_38_Zr_25_Hf_25_Ta_10_Sn_2_, 3. Ti_38_Zr_25_Hf_25_Ta_7_Sn_5_. Microstructures of various alloys in the as-cast condition. (**b**–**d**) Ti_35_Zr_27.5_Hf_27.5_Nb_5_Ta_5_, Ti_38_Zr_25_Hf_25_Ta_10_Sn_2_, Ti_38_Zr_25_Hf_25_Ta_7_Sn_5_, respectively. (**e**) TEM image of Ti_38_Zr_25_Hf_25_Ta_7_Sn_5_ alloy and the corresponding SAED patterns of matrix, BCC (**f**) and second phase, HCP (**g**).

Designing metastable *β*-Ti alloys has been one of the important achievements in Ti technological advancements of recent times. One of the effective and proven ways of designing metastable *β*-Ti alloys is using the bond order (Bo)—mean *d*-orbital energy level (Md) approach^5,8–10^. The Bo–Md approach provides a guideline for designing Ti-rich alloys from the viewpoint of controlled alloying strategy. A Bo–Md standard diagram was strategically mapped for Ti-alloys to design and fine tune various modes of deformation mechanisms such as TRIP, TWIP or dislocation slip dominant domain regions (Fig. 1a)^5,8–10^. By physically extending the room temperature martensite transformation region, this strategy has been recently used to design Ti-rich high entropy alloys (HEAs—alloys composed of ≥ 5 principal elements having each constituent 5–35 at%^11,12^). Lilensten et al.^13^ reported a successful design of Ti-rich Ti_35_Zr_27.5_Hf_27.5_Nb_5_Ta_5_ BCC-HEA, using the Bo-Md approach. Considering the alloy position in the Bo-Md diagram, the designed HEA was close to extension of the line predicting the martensite formation starting at room temperature [M_s_ = RT (Fig. 1a)]. Later, the tensile deformation and microstructure observation confirmed that deformation of the alloy (Ti_35_Zr_27.5_Hf_27.5_Nb_5_Ta_5_) was indeed assisted with TRIP. Their experimental observations confirmed the Bo-Md approach could be useful in terms of designing new Ti-rich BCC-HEAs. It should be noted however, the relevance and validity of these predictions based on this controlled alloy strategy for other alloys is not yet fully explored.

The primary objective of the present study is to investigate deformation mechanisms of new Ti-rich BCC-HEAs designed using the Bo–Md approach. Given the vast compositional space of HEAs, appropriate alloying elements must be chosen in order to exploit the required properties in terms of designing new HEAs. Here, we designed two new Ti-rich HEAs having chemical compositions Ti_38_Zr_25_Hf_25_Ta_7_Sn_5_, Ti_38_Zr_25_Hf_25_Ta_10_Sn_2_ to compare them with the Ti_35_Zr_27.5_Hf_27.5_Nb_5_Ta_5_ alloy. It is known that an addition of Sn increases *β* stability of Ti-alloys^14–16^. Furthermore, the combination of Ta and Sn alloying elements were previously reported to have advantages in terms of tuning the martensite formation temperature^15^. Therefore, in the present study, first we chose the Ti_35_Zr_27.5_Hf_27.5_Nb_5_Ta_5_ alloy^13^ as a base reference and replaced Nb with Sn, and adjusted the overall chemical compositions as Ti_38_Zr_25_Hf_25_Ta_10_Sn_2_, and Ti_38_Zr_25_Hf_25_Ta_7_Sn_5_ to make sure the alloys positions are maintained along or near the M_s_ ~ RT domain, on the Bo–Md diagram. Latter, the obtained alloys were characterized and their deformation mechanisms were systematically investigated.

## Materials and methods

Each chemical constituent element selected for the alloy making was at least 99.9 wt% purity. The alloys ingots were fabricated using vacuum arc melting process. Mechanical behaviors of all three alloys were studied using tensile deformation test. Tensile specimens having gauge dimensions 4 mm (length) × 1 mm (width) × 0.8 mm (thickness) were cut from the as-cast alloys. Tensile tests were carried out at room temperature for the constant strain-rate 10^−3^ s^−1^, deformed until fracture. Microstructural observations were performed on the fractured specimens to evaluate and access the deformation behavior of all three alloys. Microstructural observations were performed using electron back-scattering diffraction (EBSD) system operated in a field emission-scanning electron microscope (FE-SEM, FEI-Nova NanoSEM 450) and transmission electron microscope (TEM) (JEOL-JEM 2100).

## Results

**Figure 2:**
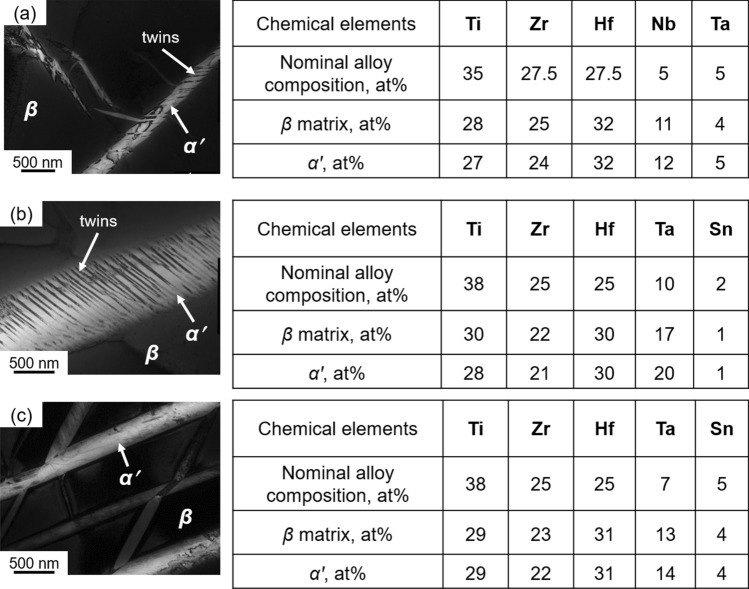
Microstructures and chemical compositions of selected alloys. TEM images of (**a**) Ti_35_Zr_27.5_Hf_27.5_Nb_5_Ta_5_, (**b**) Ti_38_Zr_25_Hf_25_Ta_10_Sn_2_, and (**c**) Ti_38_Zr_25_Hf_25_Ta_7_Sn_5_ alloys in the as-cast condition. In the insets of TEM images, ‘*β*’ indicates BCC structure, and ‘*α*′’ indicates hexagonal close packed (HCP) structure. Chemical composition of the alloy’s constituents evaluated using TEM are shown in table on the right-hand side of the respective TEM image.

Figure 1a shows schematically the standard Bo-Md diagram. Figure 1b–d shows SEM images of various alloys in the as-cast condition. The alloys showed coarse grain size, *d* ~ 220 µm in the as-cast solidified state. As-cast alloys had a peculiar needle-like second phase inside the parent coarse grains. Here, it is noteworthy that the observed needle-like structures are often referred to as Widmanstätten precipitates^2,17^, which might have formed during a fast cooling process. The as-cast alloys were characterized using TEM for identifying the constitutive phases. As all the alloys showed similar microstructural features, we choose Ti_38_Zr_25_Hf_25_Ta_7_Sn_5_ alloy as a base reference. Figure 1e shows TEM image of the Ti_38_Zr_25_Hf_25_Ta_7_Sn_5_ alloy, and Fig. 1f, g shows corresponding selected area electron diffraction (SAED) patterns. TEM characterization identified the matrix as a BCC structure, whereas the second phase particles had an HCP structure. Chemical compositions of the structural constituents of the respective alloys were estimated using TEM. Figure 2 shows the TEM images and their respective chemical composition on the right-hand side tables. It is apparent that the chemical composition of the parent BCC matrix and the HCP structures are mostly identical. This means, the HCP phase has formed by a diffusionless transformation process. Such a kind of phase transformation is referred to as displacive transformation and the resulting phase could be referred to as martensite^2^. Therefore, in the present study, we prefer to use the HCP phase as the *α*′ martensite.

**Figure 3:**
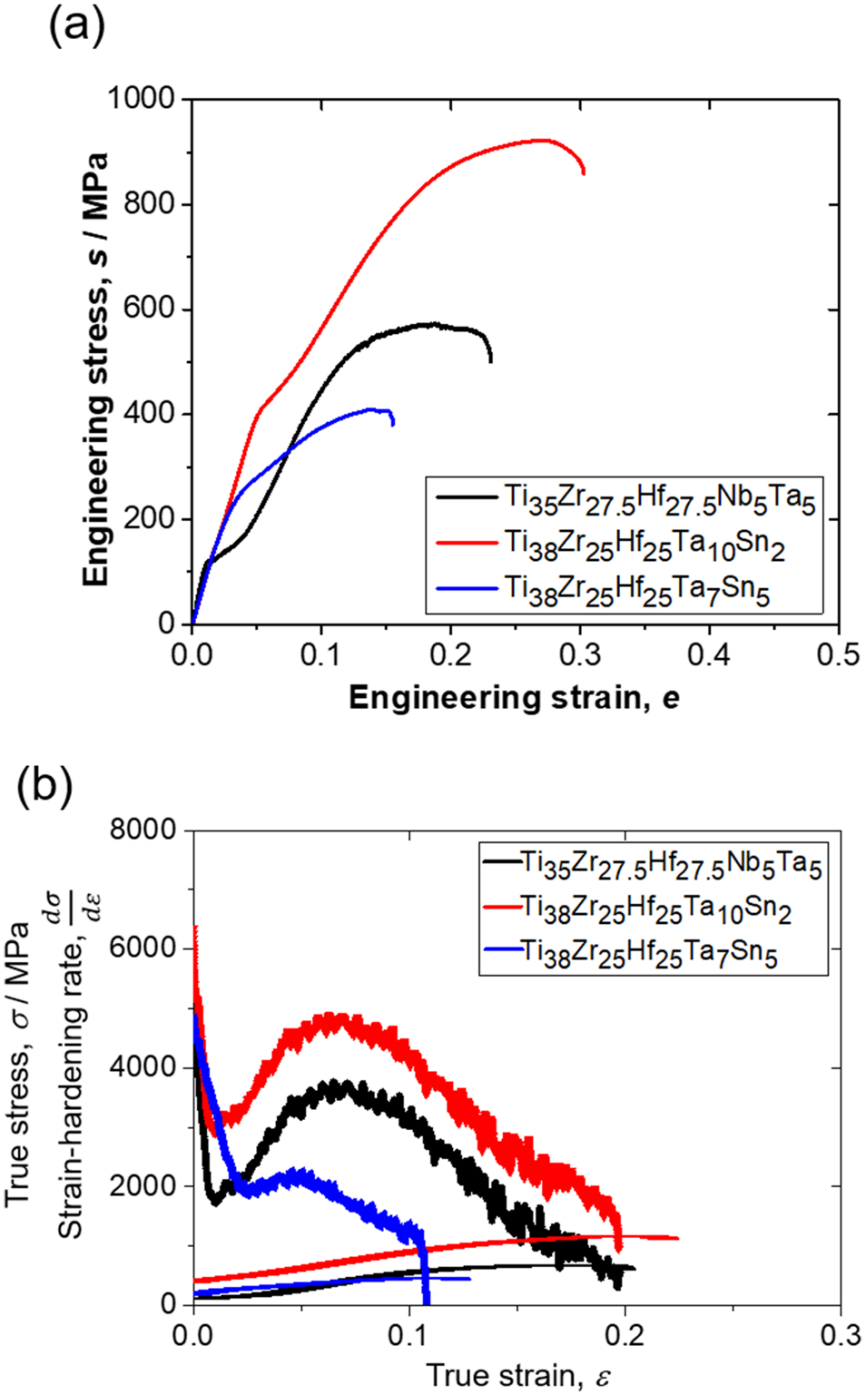
Tensile stress–strain curves and strain-hardening rate behavior of various alloys deformed at room temperature for the strain rate 10^–3^ s^−1^. (**a**) Engineering stress–strain curves. (**b**) Strain-hardening rate behavior as a function of true strain.

Mechanical behavior of the as-cast alloys was investigated using tensile deformation at room temperature. Figure 3a shows tensile engineering stress–strain curves for all three alloys. First of all, stress–strain curves indicated the alloy Ti_38_Zr_25_Hf_25_Ta_10_Sn_2_ showed the highest yield and tensile strengths, compared to the other two alloys. Further, the alloys Ti_38_Zr_25_Hf_25_Ta_10_Sn_2_ and Ti_35_Zr_27.5_Hf_27.5_Nb_5_Ta_5_ showed a similar form of yielding followed by a rapid increase in the strain-hardening, while the alloy Ti_38_Zr_25_Hf_25_Ta_7_Sn_5_ showed moderate strain-hardening. The yield strengths of Ti_38_Zr_25_Hf_25_Ta_10_Sn_2_ and Ti_35_Zr_27.5_Hf_27.5_Nb_5_Ta_5_ were 407 MPa and 121 MPa, respectively; while the maximum tensile strengths were 925 MPa and 574 MPa, respectively. Despite the same magnitude of grain size (*d* ~ 220 µm), Sn containing alloys showed higher yield strength compared with the Nb containing alloy. It has been previously reported that an addition of Sn has a strong solid solution strengthening effect in *β*-Ti alloys^18^. Here, it must be noted that Lilensten et al.^13^ reported the yield strength of Ti_35_Zr_27.5_Hf_27.5_Nb_5_Ta_5_ alloy was 540 MPa (for the grain size, *d* ~ 40 µm), compared to our lower 121 MPa (*d* ~ 220 µm). This difference can be ascribed to the effect of initial coarse grain size. In any case, the alloys showed high strain hardening behavior. For instance, the ratio of the maximum tensile strength to the yield strength gives a value of 2.2 and 4.7 times increment of tensile strength due to strain-hardening for the Ti_38_Zr_25_Hf_25_Ta_10_Sn_2_ and Ti_35_Zr_27.5_Hf_27.5_Nb_5_Ta_5_ alloys, respectively. Such an exceptionally high strain-hardening in general is uncommon for metals and alloys having a BCC matrix, due to the possibility of extensive dislocation cross-slip. Wang et al.^19,20^ reported similar form of plastic flow in Ti-rich BCC-HEAs. It is known that such a rapid strain-hardening is typically achieved due to TRIP effect^19,20^. This aspect of the stress–strain curves will be discussed in the later part of this manuscript. Meanwhile, the alloys also maintained high ductility (engineering plastic strain, *e* > 10%). Table 1 shows the summary of tensile properties of all three alloys. Figure 3b shows strain-hardening rate as a function of true strain for the three alloys. The alloys Ti_38_Zr_25_Hf_25_Ta_10_Sn_2_ and Ti_35_Zr_27.5_Hf_27.5_Nb_5_Ta_5_ showed a typical hump in the strain-hardening rate curve which clearly reflected the high strain-hardening observed in the respective stress–strain curves. On the other hand, although the alloy Ti_38_Zr_25_Hf_25_Ta_7_Sn_5_ does not show the hump as clearly as those of the other two alloys, the strain-hardening rate curve was not perfectly flat either.

**Table 1:** Summary of the tensile properties of all the three alloys.

Alloy	Yield strength, *σ*_0.2%_/MPa	Maximum tensile strength, *σ*_*m*_/MPa	Engineering plastic strain, *e* (%)
Ti_35_Zr_27.5_Hf_27.5_Nb_5_Ta_5_	121	574	19
Ti_38_Zr_25_Hf_25_Ta_10_Sn_2_	407	925	26
Ti_38_Zr_25_Hf_25_Ta_7_Sn_5_	232	409	12

**Figure 4:**
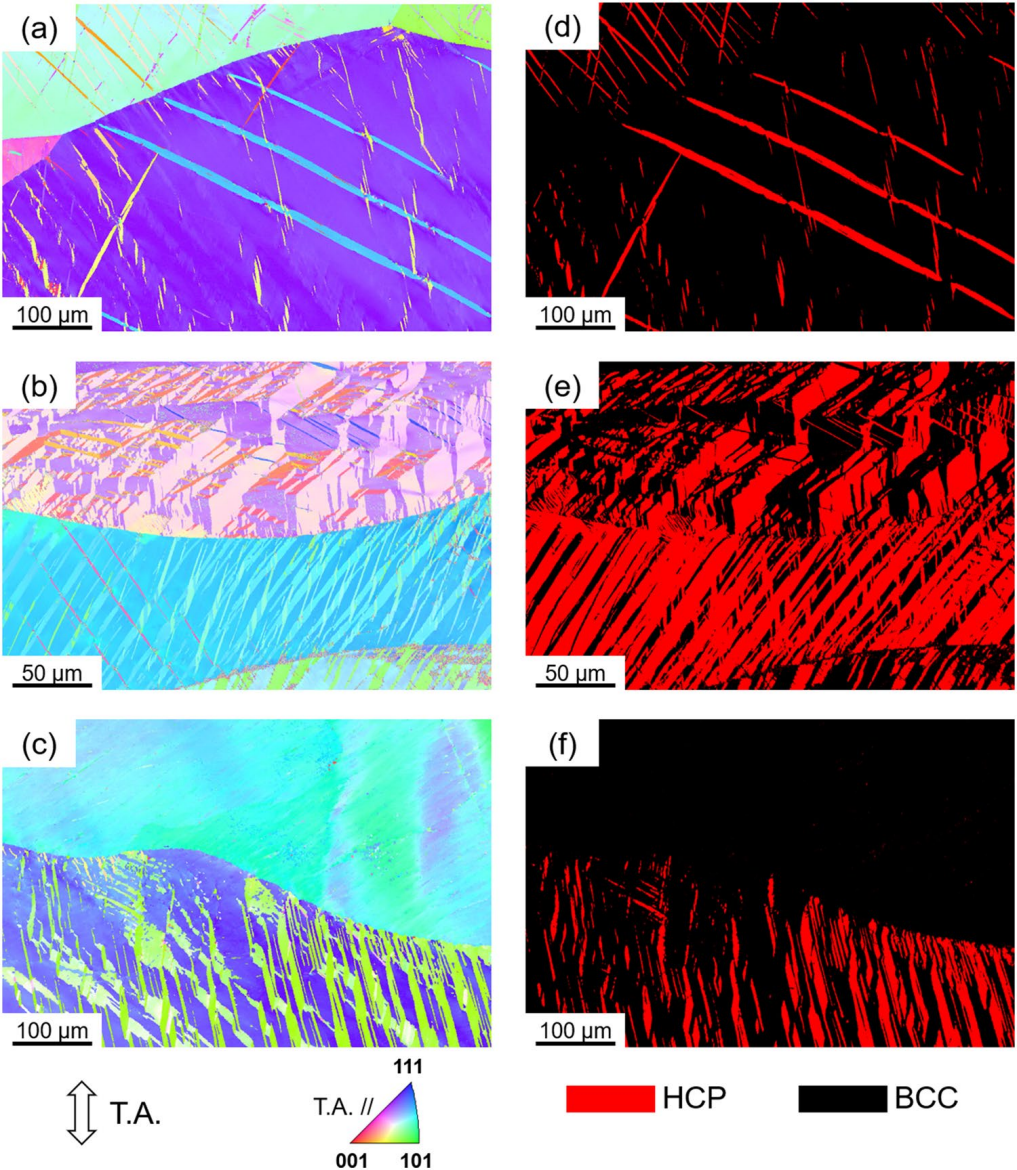
Microstructures of various alloys after tensile fracture. (**a**–**c**) EBSD IPF maps of Ti_35_Zr_27.5_Hf_27.5_Nb_5_Ta_5_, Ti_38_Zr_25_Hf_25_Ta_10_Sn_2_, and Ti_38_Zr_25_Hf_25_Ta_7_Sn_5_, respectively. Colors of the microstructures indicate crystallographic orientations parallel to the tensile axis (T.A.) according to the key stereographic triangle. (**d**–**f**) Phase maps of Ti_35_Zr_27.5_Hf_27.5_Nb_5_Ta_5_, Ti_38_Zr_25_Hf_25_Ta_10_Sn_2_, and Ti_38_Zr_25_Hf_25_Ta_7_Sn_5_, respectively. HCP and BCC phases were shown in red and black colors, respectively. Tensile axis is parallel to the vertical axis of microstructures.

Following the tensile tests, in order to understand the microstructural aspects of those stress–strain curves, the fractured specimens were carefully polished and provided for microstructure observation. Microstructures were observed on the fractured gauge surface of the tensile specimens. Figure 4 shows microstructures of all three alloys after tensile fracture. Figure 4a–c shows EBSD-IPF maps for the Ti_35_Zr_27.5_Hf_27.5_Nb_5_Ta_5_, Ti_38_Zr_25_Hf_25_Ta_10_Sn_2_, and Ti_38_Zr_25_Hf_25_Ta_7_Sn_5_ alloys, respectively. Microstructural observations revealed the BCC parent matrix formed distinct twin-like features which were characterized as the HCP, *α* phase, shown in Fig. 4d–f. It is reasonable to consider the obtained phase as the *α*′, HCP martensite, as it was formed during plastic deformation. Considering this, the microstructural observations provided a clear evidence for the occurrence of TRIP effect during tensile deformation of all the three alloys. Further, the transformed *α*′ fraction was maximum in the case of Ti_38_Zr_25_Hf_25_Ta_10_Sn_2_ alloy which showed highest tensile strength and ductility. It is noteworthy, Lilensten et al.^13^ mentioned the metastable Ti_35_Zr_27.5_Hf_27.5_Nb_5_Ta_5_ alloy has transformed into *α*″, orthorhombic martensite during tensile deformation. In the present study, after a careful EBSD characterization of the fractured tensile sample (bulk sample), we found the transformed phase has perfectly satisfied the HCP phase.

## Discussion

In the early reported studies on HEAs, the originally considered equiatomic solid solution alloys were predominantly stable during deformation at ambient and elevated temperatures^12,21^. However, the emergence of non-equiatomic HEAs have shown new possibilities in terms of exploring mechanical properties and phase stability of these alloys. Many of the reported non-equiatomic HEAs were metastable, and facilitate phase transformation under externally applied stress, thus resulting in the TRIP effect^22,23^. TRIP assisted deformation of FCC-HEAs and BCC-HEAs have been actively studied recently^13,24–27^. Li et al.^26^ showed metastable FCC based-HEA predominantly deformed by TRIP effect maintaining both strength and ductility. Similarly, Huang et al.^27^ reported a series of Ta_x_TiHfZr BCC-HEAs. They found that a decrease in the Ta percentage increased the propensity to the BCC to HCP phase transformation during deformation. Although we chose non-equiatomic concentrations (i.e., Ti_35_Zr_27.5_Hf_27.5_Nb_5_Ta_5_, Ti_38_Zr_25_Hf_25_Ta_10_Sn_2_, and Ti_38_Zr_25_Hf_25_Ta_7_Sn_5_), our alloy compositions were guided by the Bo-Md approach. Based on the position of the alloys on the Bo-Md diagram, all the alloys showed TRIP effect.

In the present study, as the alloy Ti_38_Zr_25_Hf_25_Ta_10_Sn_2_ showed high strain-hardening, high tensile strength, and ductility simultaneously, further discussions are generalized using these observations. Basically, the advantages of TRIP effect are manifold. Firstly, as both the phases have same chemical composition, the transformed HCP phase may have high strength compared to the matrix BCC phase, due to limited slip systems of HCP phase. Based on this, the matrix *β* could be soft compared to the *α*′. The transformed *α*′ having high hardness values compared to the *β* phase was previously reported^28,29^. Therefore, it is to note that the prior metastable, soft *β* phase transforms into a relatively hard *α*′ phase. Second, the transformed *α*′ generates a distinct phase boundary with the parent *β* matrix. A phase boundary is a structural defect analogous to a grain boundary in terms of continuity of crystallographic planes is broken. Consequently, the incidence of phase boundary due to the formation of *α*′ dynamically refines the effective *β* grain size and decreases the mean free path for the dislocations slip, one of the primary carriers of plastic deformation. This is technically referred to as “dynamic Hall–Petch” effect^1,30,31^. Furthermore, the presence of phase boundaries induces inhomogeneous deformation, in order to ensure compatibility of the applied stress throughout the deformation sample. As a result of this, excess dislocations are generated (also referred to as geometrically necessary dislocations (GNDs)) along the phase boundary^32^. All these processes generate immense long range stress field from the phase boundary into the parent matrix, which also may contribute to strengthening^33^. It should be noted however, high stress concentration in a local region can lead to immediate fracture. In order to avoid such a pre-mature failure, stress concentration must be relaxed by activating additional slip/twin systems or through the growth of the transformed *α*′ martensite, which accounts for additional plasticity. Consequently, TRIP assisted deformation processes maintain both high strength and ductility.

**Figure 5:**
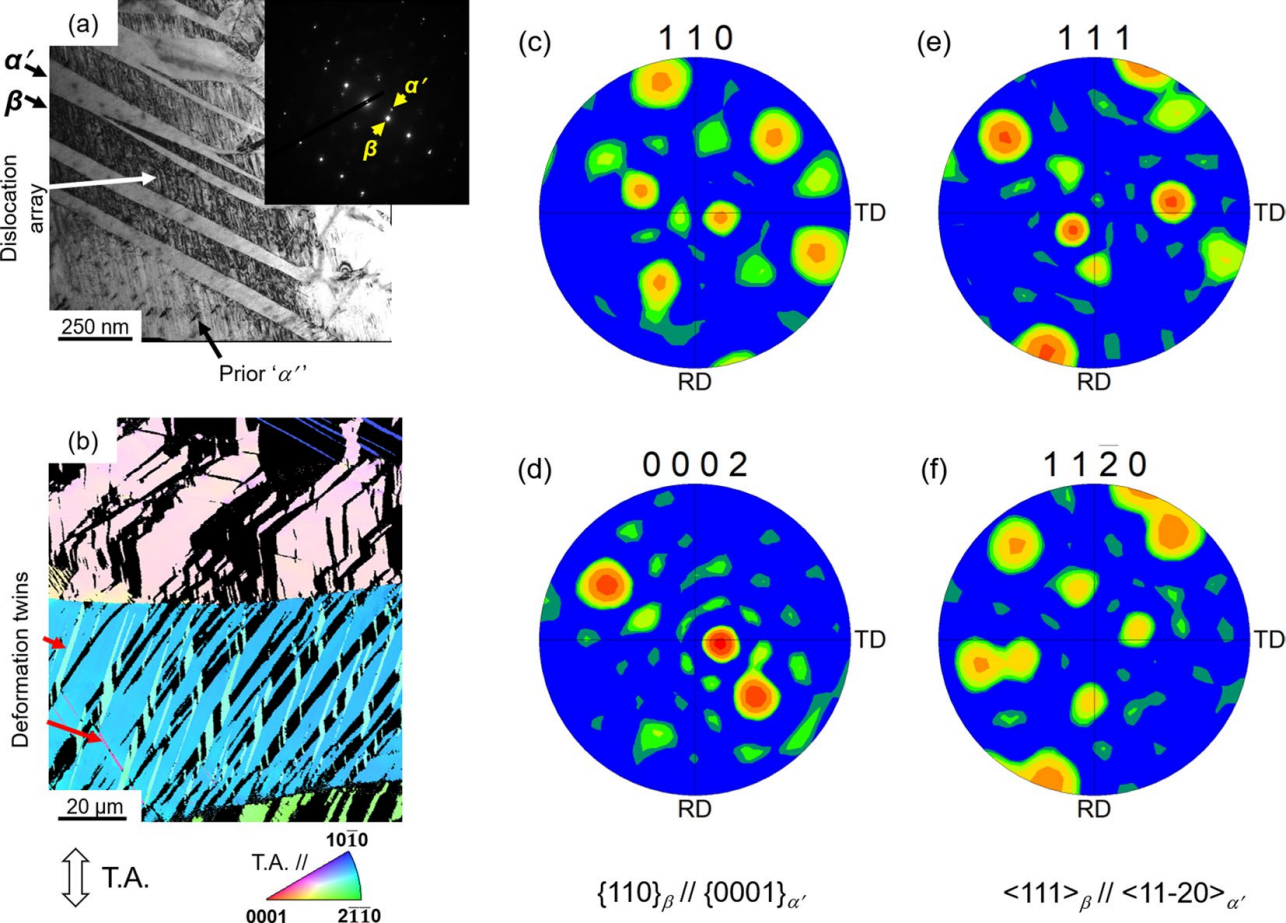
Microstructures of Ti_38_Zr_25_Hf_25_Ta_10_Sn_2_ alloy after tensile fracture. (**a**) TEM image. SAED pattern is shown in the inset. (**b**) EBSD-IPF map of HCP phase. BCC phase is highlighted in black. Colors of the microstructure indicate crystallographic orientations parallel to the tensile axis (T.A.) according to the key stereographic triangle. Tensile axis is parallel to the vertical axis of microstructures. Pole figure (PF) plots of Ti_38_Zr_25_Hf_25_Ta_10_Sn_2_ alloy corresponding to the microstructure shown in (**b**) after tensile fracture. (**c**) 110, (**d**) 0002, (**e**) 111, and (**f**) 11–20.

Further investigations were focused on the substructure of the deformed specimens. Figure 5a shows TEM image of Ti_38_Zr_25_Hf_25_Ta_10_Sn_2_ alloy after tensile fracture. TEM image revealed the typical composite-like structure featuring *β* matrix and transformed *α*′. It was also evident that the *β* phase deforms by dislocation slip, confirmed from the observed array of dislocations. It is noteworthy that the *α*′ once formed is not isolated from the overall plastic deformation and may also deform by twinning or dislocation slip. Figure 5b shows EBSD-IPF map of the Ti_38_Zr_25_Hf_25_Ta_10_Sn_2_ alloy after tensile fracture. Deformation twins were observed inside the transformed *α*′. Here, deformation twins were recognized as regions of sharp orientation difference in the HCP phase, pointed by arrow marks in the Fig. 5b. As the prior *β* phase undergoes phase transformation (generates transformation strain) and dislocation slip, the transformed *α*′ deformed by twinning. All these simultaneously occurring processes significantly harden the alloy and facilitate ductility also, as was reflected in the stress–strain curve (Fig. 2). Furthermore, as the present Ti-rich BCC-HEAs belong to a new class of TRIP-HEAs, understanding crystallographic orientation relationship (OR) between the parent *β* matrix and the transformed *α*′ is highly desired. Figure 5c–f shows the pole figure (PF) plots of the Ti_38_Zr_25_Hf_25_Ta_10_Sn_2_ alloy corresponding to the EBSD-IPF map shown in Fig. 5b. Figure 5c,d shows a set of crystallographic OR between *β* and transformed *α*′, (1 1 0)_*β*_//(0 0 0 1)_*α*′_. Further, Fig. 5e,f shows the PF plots also indicated [1 − 1 1]_*β*_//[1 1 − 2 0]_*α*′_. That is, the transformation of *β* to *α*′ showed specific variants selection. These variants belongs to the classical Burgers orientation relationship (BOR) between *β* and *α* phases, which were most commonly observed in the Ti-alloys^34–38^. In the sense, it is worthy emphasizing that the transformation of *β* to *α*′ of Ti-rich HEAs follow the classical BORs. These orientation relationships ensure two phases the best possible fit at the phase boundary, so that the two phases maintain low energy interface which facilitate low nucleation or growth barrier during transformation^2,3^. Furthermore, correspondence with the classical BORs, the transformation mechanism of *β* to *α* of Ti and Zr alloys has been widely regarded as the homogeneous lattice distortion mechanism^2,39^. Based on the similarities of chosen BOR between Ti-rich HEAs and Ti-alloys, from our standpoint it is seemingly reasonable to suggest similar transformation mechanism would prevail for the transformation of *β* to *α*′ in the Ti-rich BCC-HEAs.

Finally, based on the experimental observations, the alloys designed using the Bo-Md approach were found to be extremely attractive both in terms of deformation behavior and mechanical properties. Controlling the microstructure, especially decreasing the initial grain size using appropriate thermo-mechanical processing route might result in a remarkable increment of strength and ductility^40–42^. Further optimization of microstructural parameters is being done and will be reported in our future article.

## Conclusions

To conclude, new Ti-rich HEAs designed using theoretical predictions of the Bo-Md approach were systematically investigated on alloys located at different positions along the reference line of M_s_ = RT on the standard Bo-Md diagram. The designed alloys showed exceptionally high strain-hardening and ductility. Microstructural characterization after tensile deformation confirmed the transformation of *β* to *α*′, indicating TRIP assisted deformation mechanism. The evaluated experimental observations successfully reproduced the TRIP effect confirming controlled alloying strategy using the Bo-Md approach could be effectively applied for designing new Ti-rich HEAs having various alloy compositions that can maintain high tensile strength and ductility. On the other hand, transformation of *β* to *α*′ showed strong variant selection yet followed the classical Burgers orientation relationship. Crystallographic analysis provided insights into the transformation mechanism of *β* to *α*′ in Ti-rich HEAs.
